# A high‐fat diet impairs mitochondrial biogenesis, mitochondrial dynamics, and the respiratory chain complex in rat myocardial tissues

**DOI:** 10.1002/jcb.27068

**Published:** 2018-09-01

**Authors:** Dan Chen, Xia Li, LiTing Zhang, Mei Zhu, Ling Gao

**Affiliations:** ^1^ Department of Endocrinology Shandong Provincial Hospital Affiliated to Shandong University, Shandong Provincial Key Laboratory of Endocrinology and Lipid Metabolism, Institute of Endocrinology and Metabolism, Shandong Academy of Clinical Medicine Jinan China; ^2^ Department of Electrocardiographic Shandong Provincial Hospital affiliated to Shandong University Jinan China; ^3^ Department of Ultrasound Shandong Provincial Hospital affiliated to Shandong University Jinan China

**Keywords:** energy metabolism, fission, fusion, heart failure, high‐fat diet, mitochondria, proteomics

## Abstract

A high‐fat diet (HFD) has been associated with heart failure and arrhythmias; however, the molecular mechanisms underlying these associations are poorly understood. The mitochondria play an essential role in optimal heart performance, most of the energy for which is obtained from the oxidation of fatty acids. As such, chronic exposure to excess fatty acids may cause mitochondrial dysfunction and heart failure. To investigate the effects of a HFD on the mitochondrial function in the myocardium, 40 male rats were randomly divided into two groups and fed with either a normal diet or a HFD for 28 weeks. The myocardial lipid content, cardiac parameters and function, and mitochondrial morphology and function were evaluated. The expression of a number of genes involved in mitochondrial dynamics was measured using quantitative polymerase chain reaction and Western blot analyses. Proteomic analysis was also performed to identify the proteins affected by HFD treatment. Significant fat deposition in the myocardia, cardiac hypertrophy, and cardiac dysfunction were all observed in HFD‐treated rats. Electron microscopy showed abnormal mitochondrial density and morphology. In addition, abnormal expression of genes involved in mitochondrial dynamics, decreased mitochondrial DNA copy numbers, reduced complex I‐III and citrate synthase activities, and decreased mitochondrial respiration were observed in HFD‐treated rats. High performance liquid chromatography showed downregulated adenosine triphosphate (ATP) and adenosine diphosphate levels and an increased adenosine monophosphate (AMP)/ATP ratio. Proteomic analysis confirmed the alteration of mitochondrial function and impaired expression of proteins involved in mitochondrial dynamics in HFD‐treated rats. Mitochondrial dysfunction and impaired mitochondrial dynamics play an important role in heart dysfunction induced by a HFD, thus presenting a potential therapeutic target for the treatment of heart disease.

## 1 INTRODUCTION

The adult heart obtains most of its energy from the oxidation of fatty acids (FAs), of which there are high levels in patients who consume a high‐fat diet (HFD).^1^, ^2^, ^3^ In the cytoplasm of cardiomyocytes, 80% of FAs are directly delivered to the mitochondria for β‐oxidation and 20% are esterified to triglyceride for temporary storage in cytoplasmic lipid droplets.^1^, ^3^, ^4^ While FAs are the major energy source of the myocardium, long‐term exposure to free FAs may result in fat accumulation in cardiomyocytes and around the heart, leading to lipotoxicity. In addition, chronic exposure to excess fats in the circulation can have a detrimental effect on the heart due to the accumulation of toxic metabolic derivatives, such as reactive oxygen species (ROS) and ceramides, which can cause myocyte dysfunction and death via activation of certain signaling cascades.^5^, ^6^, ^7^, ^8^

Consuming a HFD is known to be a major cause of obesity, which is strongly associated with cardiovascular diseases, such as coronary heart disease (CHD) and heart failure (HF).^9^ Recently, increasing evidence has suggested that dietary fat intake can influence the development of HF.^9^

As highly dynamic organelles, mitochondria undergo fission to generate fragmented mitochondria or fusion to form an interconnected elongated morphology.^10^ These two processes are tightly regulated by specific fusion and fission proteins so as to maintain a dynamic balance, which can subsequently influence many essential biological processes, including cell division, apoptosis, autophagy, and metabolism.^11^ Optimal cardiac mitochondrial fitness is disrupted in adult cardiomyocytes after the ablation of mitochondrial fission and fusion proteins, suggesting roles for these proteins in the mitophagic process, ensuring mitochondrial quality control in the adult heart.^12^ The role of mitochondria in the development of heart dysfunction has been extensively studied^13^; however, the detrimental effects of a HFD on mitochondria are not fully understood. In the current study, we investigate the effects of a HFD on mitochondrial function and dynamics, as well as in regard to cardiac dysfunction.

## 2 MATERIALS AND METHODS

### 2.1 Animal model

Forty male Sprague‐Dawley rats (body weight: 170 to 190 g, 6 weeks old) purchased from Vital River Laboratory Animal Technology Co Ltd (Beijing, China) were used in this study. All animals received proper care, and all animal experiments were approved and supervised according to the guidelines of the Animal Care and Use Committee of Shandong Provincial Hospital. The rats were kept in the same facility (20 ± 2°C, 60 ± 5% humidity, 12/12 hours light‐dark cycle) with unlimited access to food and water. After one week of adaption to the environment, the rats were randomly divided into two groups: (1) the control group (n = 20) was fed a diet containing 10% fat, 70% carbohydrate, and 20% protein; (2) the HFD group (n = 20) was fed a diet containing 5.24 kcal/g, 60 kcal% fat, 20 kcal% carbohydrate, and 20 kcal% protein (Research Diets, D12492). Rats were killed by the end of the 28th week, and tissues and blood were harvested for subsequent experiments.

### 2.2 Echocardiography

At week 28, rats were anaesthetized using 3% pentobarbital sodium (0.1 mL/100 mg) and their cardiac function and ventricular dimensions were evaluated by transthoracic echocardiography using a GE vivid E9 equipped with a 13‐MHz phased‐array transducer (General Electric Company, Boston, MA). All rat hearts were recorded at the level of the papillary muscle in 2D and M‐mode. The parameters were determined for three cardiac cycles and averaged. The measurements were taken by the same person, who was blinded to the study design.

### 2.3 Cardiac dissection

After 28 weeks of feeding, the hearts were rapidly excised from the rats under anesthesia (3% pentobarbital sodium). The left ventricles (LVs) were quickly cut into three sections, which were then fixed in 2.5% glutaraldehyde and 1% paraformaldehyde for electron microscopy, immersed in RNAiso Plus (TakaRa, Dalian, China) for RNA isolation, or frozen in liquid nitrogen for follow‐up experiments. The body weight, heart weight, and LV weight of each rat were measured and normalized by the length of the thighbone.

### 2.4 Blood and myocardial lipid concentration assay

Triglycerides (TG) and total cholesterol (TC) levels in the plasma were measured using an Olympus AU 600 auto analyzer (Olympus, Japan). Myocardial TG and TC were determined using an assay kit according to the manufacturer's instructions (Applygen, Beijing, China). The myocardial FFA content was determined with a free fatty acid (FFA) quantification kit (BioVision, San Francisco, CA).

### 2.5 Electron microscopy

The left ventricular anterior wall was obtained from the freshly excised hearts, fixed in 2.5% glutaraldehyde and 1% paraformaldehyde at 4°C for 24 hours and then washed with 0.1M phosphate‐buffered solution at 4°C, stained in 2% osmium tetroxide, embedded in resin, and sectioned. After washing twice, the tissues were postfixed with a 1% OsO_4_‐buffered solution (pH 7.4) for 90 minutes. Samples were then dehydrated with serial ethanol and propylene oxide treatments and were embedded in Poly/EM Bed812 (Polysciences, Warrington, PA) embedding medium. The resin was then polymerized in a vacuum drying oven at 60°C for 48 hours. Tissues were sectioned using an EM Ultramicrotome LKB‐2088 and stained with 1% toluidine blue. Ultrathin sections were then double‐stained with uranyl acetate and lead citrate and examined with a Hitachi H‐7600 electron microscope (Hitachi, Tokyo, Japan).

### 2.6 Isolation of mitochondria

A total of 100 mg of the fresh LV was quickly removed and washed with ice‐cold 1× phosphate buffered saline (PBS) buffer (pH 7.4). The LVs were minced with scissors on ice and briefly centrifuged at 12 000 rpm and 4°C to remove the 1× PBS. The pellets were resuspended in 1 mL mitochondrial isolation buffer (225 mM mannitol, 75 mM sucrose, 2 mM K_2_HPO_4_·3H_2_O, 1 mM ethylene glycol‐bis(β‐aminoethyl ether)‐N,N,N',N'‐tetraacetic acid (EGTA), pH 7.2), homogenized, and centrifuged at 1300 *g* at 4°C for 5 minutes. The supernatant was then mixed with 3 mL 15% vol/vol procell (Beijing Solarbio Science Technology Co, Beijing, China) and centrifuged at 36 500*g* for 17 minutes at 4°C. After discarding the supernatant, the brown pellet containing the mitochondria was gently resuspended in 1 mL mitochondrial isolation buffer and centrifuged at 10 000*g* and 4°C for 10 minutes. The pellets were resuspended in 1 mL mitochondrial isolation buffer and centrifuged at 8 000*g* for 10 minutes at 4°C. The final brown pellets of mitochondria were immediately used for measurement of mitochondrial respiratory chain enzymatic activities or stored at −80°C for determination of protein production. To extract the mitochondrial proteins, the disruption of isolated mitochondria was achieved by repeated freeze/thaw cycles. The mitochondrial protein concentration was determined using an ultraviolet spectrophotometer.

### 2.7 Quantitative polymerase chain reaction

Total RNA was extracted from the LV using RNAiso Plus (TakaRa, China). Next, 1000 ng of total RNA was used for cDNA synthesis using the PrimeScript cDNA Synthesis Kit (Takara, Japan). Quantitative real time polymerase chain reaction (RT‐PCR) reactions were performed using the ABI PRISM 7500 Sequence Detection System (Applied Biosystems) with the primers listed in Table 1. The messenger RNA (mRNA) concentrations of Mfn1, Mfn2, Opa1, Drp1, and Fis1 were determined, and their relative expression levels were quantified using the $2^{{-\Delta \Delta C}_{t}}$ method.

**Table 1:** Primers used in quantitative polymerase chain reaction

Gene	Forward primer (5′→3′)	Reverse (5′→3′)	Amplicon size, bp	GenBank accession
*MFN1*	CCTTGTACATCGATTCCTGGGTTC	CCTGGGCTGCATTATCTGGTG	143	NM_138976
*MFN2*	GATGTCACCACGGAGCTGGA	AGAGACGCTCACTCACTTTG	136	NM_130894
*OPA1*	CAGCTGGCAGAAGATCTCAAG	CATGAGCAGGATTTTGACACC	107	NM_133585
*DLP1*	CGTAGTGGGAACTCAGAGCA	TGGACCAGCTGCAGAATAAG	120	NM_053655
*FIS1*	CATCCGTAGAGGCATCGT	TGTCAATCAGGCGTTCCA	197	NM_001105919.1
*β‐Actin*	CTAAGGCCAACCGTGAAAAGA	CCAGAGGCATACAGGGACAAC	100	NM_031144
*COXIV*	TAATTCGAGCTGAACTAGGAC	TACAAGTCAGTTCCCGAAGC	238	NC_001665.2
*RPL4*	CACGCAAGAAGATTCATCGC	AACAATCTTCTCCGATTTGGC	173	NM_022510.1

Total DNA was extracted using a TIANamp Genomic DNA Kit (Tiangen, Beijing, China) and 40 ng of DNA was used for the quantitative PCR analysis. Nuclear targeting sequences were determined using nuclear gene ribosomal protein L4 (RPL4) primers and mitochondrial targeting sequences were quantified using cytochrome *c* oxidase subunit 1 (COXIV) primers. The mitochondrial DNA copy number was quantified using the $2^{-{\Delta \Delta C}_{t}}$ method.

### 2.8 Western blot analysis

The left ventricular tissues and isolated mitochondrial samples were homogenized in RIPA buffer supplemented with protease and phosphatase inhibitors on ice to obtain total protein samples. Protein concentrations were determined using an enhanced BCA protein assay kit (Beyotime, Shanghai, China). The soluble lysates (90 µg total protein per 10 µg mitochondrial protein) were separated by 10% sodium dodecyl sulfate polyacrylamide gel electrophoresis before being transferred to polyvinylidene difluoride (PVDF) membranes and blocked overnight with 5% (wt/vol) milk at 4°C. The PVDF membranes were then incubated overnight with primary antibodies for Mfn1 (Abcam, Cambridge, UK; #57602; 1:1000 dilution), Mfn2 (Abcam; #56889; 1:1000 dilution), Opa1 (Cell Signaling Technology, Massachusetts, MA; #80471; 1:2000 dilution), Drp1 (Cell Signaling Technology; #611113; 1:1000 dilution), phospho‐Drp1 (Cell Signaling Technology; #4494s, 1:1000 dilution), Fis1 (Proteintech, Chicago, IL; #10956‐1‐AP; 1:500), glyceraldehyde 3‐phosphate dehydrogenase (GAPDH) (Abcam; #9485; 1:7500 dilution), or COXIV (Proteintech; #11242‐1‐AP; 1:1000 dilution) at 4°C, followed by incubation with an horseradish peroxidase‐conjugated secondary antibody (1:5 000 dilution) at room temperature for 1 hour. The bands were visualized using an Alpha Fluorchem Q Imaging analysis system (Cell Biosciences, Santa Clara, CA) and quantified via scanning densitometry. Either GAPDH or COXIV served as the loading control for the Western blot experiments.

### 2.9 Adenine nucleotide analysis

Frozen LVs were transferred to ice‐cold 0.6M HClO_4_ (4 mL/g), and the tissue was immediately homogenized and centrifuged (10 000*g*, 4°C, 10 minutes). The supernatant was neutralized with an equal volume of 1M Na_2_HPO_4_ and centrifuged again at 10 000*g* and 4°C for 10 minutes. The supernatant was filtered through a 0.22 µm filter. Next, 50 µl aliquots were analyzed using a high performance liquid chromatography (HPLC) method with a Beckman C18 column (5 μm, 250 × 4.6 mm). Analytes were isocratically eluted using 96% 0.05M KH_2_PO_4_ (pH 6.5) and 4% methanol for 30 minutes. Concentrations of adenosine triphosphate (ATP), adenosine diphosphate (ADP), and adenosine monophosphate (AMP) were determined at 254 nm using an external standard method for quantification. The energy charge was defined as (ATP + ADP/2)/(ATP + ADP + AMP).

### 2.10 Mitochondrial respiratory chain enzymatic activities

The activities of mitochondrial complexes I‐III were measured as previously described^14^ with modifications. The LV mitochondria were isolated as described above. Complex I activity was determined by measuring the oxidation of nicotinamide adenine dinucleotide (NADH) at 340 nm in potassium phosphate buffer (0.5M, pH 7.5), 50 mg/mL fatty acid free (FAF)‐BSA, 10 mM KCN, 10 mM NADH, and 10 mM ubiquinone, which was added last to start the reaction. Complex II activity was determined by measuring the reduction of the artificial electron acceptor L2, 6‐dichlorophenolindophenol at 600 nm in potassium phosphate buffer (0.5M, pH 7.5), 50 mg/mL FAF‐BSA, 10 mM KCN, 400 mM succinate, and 1 mM malate, which was added last to start the reaction. Complex III activity was measured by the reduction of cytochrome *c* at 550 nm in a reaction mixture of potassium phosphate buffer (0.5M, pH 7.5), oxidized cytochrome *c*, 10 mM KCN, 5 mM EDTA (pH 7.5), 2.5% (vol/vol) Tween‐20, and 10 mM decylubiquinol, which was added last to start the reaction. Citrate synthase activity was determined as described by Spinazzi et al.^14^ The reduction of acetyl‐CoA was measured in the presence of oxaloacetate at 412 nm in a reaction mixture consisting of 200 mM Tris (pH 8.0) with 0.2% (vol/vol) Triton X‐100, 0.2 mM dinitrobenzoic acid (DTNB), and 10 mM acetyl‐CoA. The reaction was started by the addition of 10 mM oxaloacetic acid.

### 2.11 Mitochondrial proteomics

The myocardial mitochondrial proteins from either the control (n = 10) or HFD groups (n = 10) were pooled. According to the manufacturer's instructions, 10 µg of mitochondrial protein from each sample was trypsin digested and labeled using the iTRAQ Reagent Multi‐Plex Kit (Sciex, Canada), and the mitochondrial proteome was analyzed using an AB SCIEX TripleTOF 5600+ system (Ab Sciex, Foster City, CA).

### 2.12 Statistical analysis

Data are expressed as mean ± standard deviation. Data from the control and HFD groups were compared using the *t* test. A *P*‐value of less than 0.05 was considered significant.

## 3 RESULTS

### 3.1 HFD causes excess weight gain and heart dysfunction in rats

**Figure 1:**
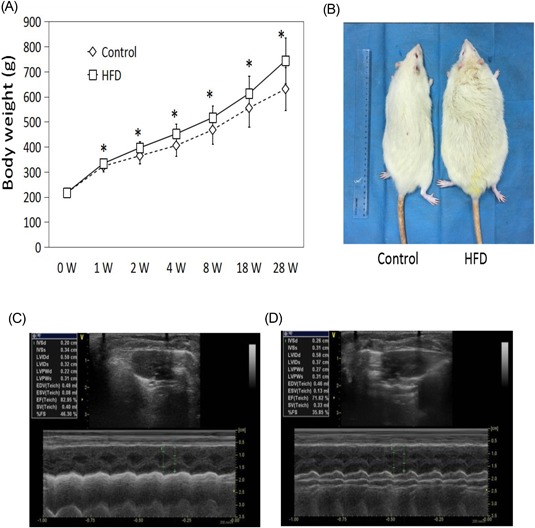
HFD causes excess weight gain and heart dysfunction in rats. (A) Rats from the HFD group showed a significant increase in body weight compared with the rats from the control group. (B) Compared to the normal group, the bodyweight in the HFD group was higher. (C) Control‐group transthoracic echocardiography. (D) HFD‐group trans thoracic echocardiography. Diagnostic transthoracic echocardiography showed left ventricular dilatation, systolic dysfunction, and interventricular septal thickness. HFD, high‐fat diet

Rats from the HFD group began to show a significant increase in body weight compared with the rats from the control group after only 1 week (Figure 1A). The difference in body weight continued to increase as the feeding progressed. By week 28, the average weight of the HFD group was 744.1 ± 91.5 g, while that of the control group was 630.9 ± 85.4 g (Figure 1A).

Cardiac function and ventricular dimensions of both the control and HFD rats were measured by transthoracic echocardiography after the full 28 weeks of treatment (Figure 1C,D). As shown in Table 2, HFD rats showed higher measurements in the following categories compared with the control group: interventricular septal thickness at diastole (IVSd; 2.48 ± 0.08 vs 2.16 ± 0.15 mm, *P* < 0.05), left ventricular internal diameter end systole (LVIDs; 3.84 ± 0.52 vs 3.18 ± 0.32 mm, *P* < 0.05), left ventricular posterior wall end diastole (LVPWd; 2.52 ± 0.25 vs 2.10 ± 0.10 mm, *P* < 0.05), and left ventricular posterior wall end systole (LVPWs; 3.30 ± 0.25 vs 2.82 ± 0.36 mm, *P* < 0.05). The HFD group also showed decreases in the ejection fraction (EF; 73.3 ± 2.0 vs 82.7 ± 5.3%, *P* < 0.05) and fractional shortening (FS; 37.3 ± 1.7 vs 46.5 ± 5.1%, *P* < 0.05). No significant differences were observed for interventricular septal thickness at systole (IVSs), left ventricular internal diameter end diastole (LVIDd), end‐diastolic volume (EDV), end‐systolic volume (ESV), or stroke volume (SV) (Table 2). These results suggested impaired heart function in the rats from the HFD group.

**Table 2:** HFD caused cardiac hypertrophy, increased IVsd, LVDs, LVPW and LVPWs level, and decreased the EF and FS level

Group	Number	IVSd, (mm)	IVSs, (mm)	LVIDd, (mm)	LVIDs, (mm)	LVPWd, (mm)	LVPWs, (mm)	EDV, (mL)	ESV, (mL)	EF, (%)	FS, (%)	SV, (mL)
CON28w	5	2.16 ± 0.15	3.22 ± 0.14	5.94 ± 0.36	3.18 ± 0.32	2.1 ± 0.1	2.82 ± 0.36	0.49 ± 0.08	0.08 ± 0.03	82.67 ± 5.27	46.46 ± 5.07	2.16 ± 0.15
HFD28W	5	2.48 ± 0.08^*^	3.1 ± 0.32	6.18 ± 0.88	3.84 ± 0.52^*^	2.52 ± 0.25^*^	3.3 ± 0.25^*^	0.56 ± 0.24	0.15 ± 0.06	73.28 ± 1.98^*^	37.3 ± 1.7^*^	2.48 ± 0.08^*^

CON, control; EDV, end‐diastolic volume; EF, ejection fraction; FS, fractional shortening; ESV; end‐systolic volume; HFD, high‐fat diet; IVSd, interventricular septal thickness at diastole; IVSs, interventricular septal thickness at systole; LVIDd, left ventricular internal diameter end diastole; LVIDs, left ventricular internal diameter end systole; LVPWd, left ventricular posterior wall end diastole; LVPWs, left ventricular posterior wall end systole; SV, stroke volume.

^*^ Significant changes compared with the control, *P *< 0.05.

### 3.2 HFD induces cardiac hypertrophy and fat accumulation in myocardia

**Figure 2:**
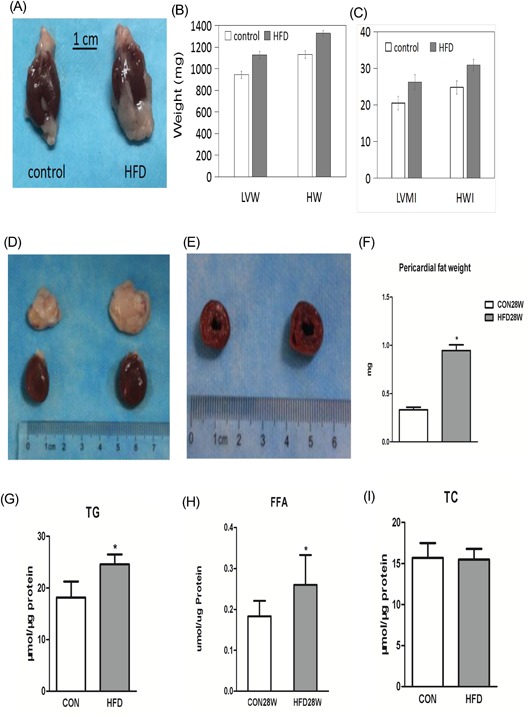
HFD induces cardiac hypertrophy and fat accumulation in myocardia. (A) Heart appearance (control vs HDF). (B) Compared with the control, the left ventricle mass and whole heart mass were increased. (C) The left ventricle mass index and heart mass index in the HDF group were increased. (D) Compared to the control, epicardial adipose tissue was increased. (E) The left ventricular cavity and left ventricular wall thickness of the HDF group were increased. (F) Compared to the control, the weight of the epicardial adipose tissue was increased. (G‐I) TG and FFA levels of the HDF group were increased (*P* < 0.5); TC showed no significant change. FFA, free fatty acid; HFD, high‐fat diet; TC, total cholesterol

The hearts of both the control and HFD rats were dissected and photographed (Figure 2A). LV weight and whole heart weight were determined to evaluate for signs of cardiac hypertrophy (Figure 2B). The average left ventricle mass (LVM) and heart mass (HM) of HFD rats were 1128 ± 31 and 1327 ± 26 mg, respectively, which were significantly higher than those of the control group (944 ± 33 and 1130 ± 37 mg, respectively, *P* < 0.05; Figure 2B). After normalization to the length of the thighbone, the resulting left ventricle mass index (LVMI) (26.2 ± 2.1 vs 20.5 ± 1.9 mg/mm) and heart mass index (30.9 ± 1.6 vs 24.8 ± 1.8 mg/mm, *P* < 0.05) still showed significant differences between the HFD and control groups (Figure 2C). Moreover, compared to the control group, the weight of the epicardial adipose tissue was increased (*P* < 0.05; Figure 2D,F).

**Figure 3:**
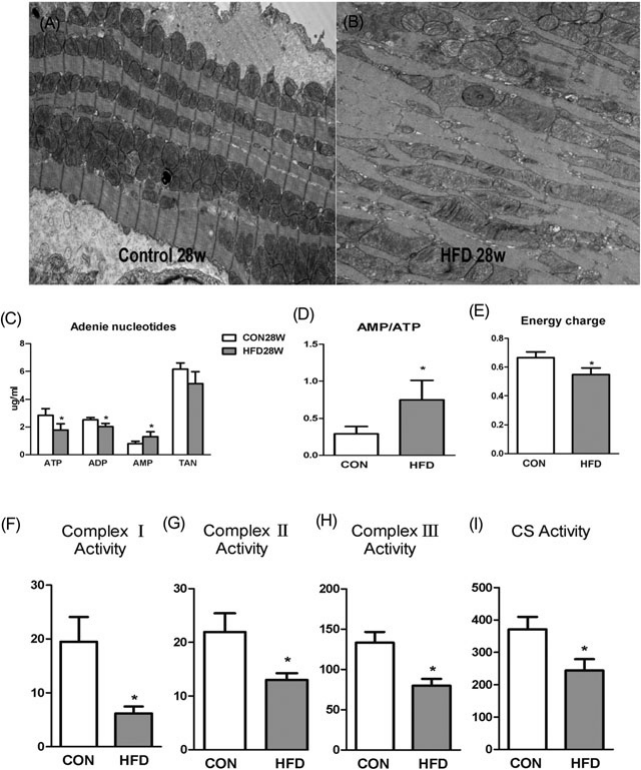
Changes in mitochondrial morphology and mitochondrial activity in the HFD and control groups. (A,B) The structure of the HFD group myocardia showed obscured striation and partial and even complete disappearance of the z line. Ultrastructural analysis of the mitochondria showed that mitochondrial density was noticeably lower in the HFD group myocardia, with a greater variation in size. Moreover, they appeared to be bloated and had fewer cristae structures in the inner membrane. (C‐E) ATP and ADP concentrations in the myocardia of the HFD group were significantly lower than those of the control group. Conversely, the AMP concentration was significantly higher in the HFD group than in the control group. No significant differences existed between the control and HFD groups in regard to TAN contents. The HFD group had a significantly higher AMP/ATP ratio and a lower energy charge relative to the control group. (F‐I) Mitochondrial complex enzyme activities were decreased dramatically in the HFD group. ADP, adenosine diphosphate; ATP, adenosine triphosphate; AMP, adenosine monophosphate; FFA, free fatty acid; HFD, high‐fat diet; TAN, total adenine nucleotide; TC, total cholesterol

The myocardial fat contents were also compared between the HFD and control groups. Not surprisingly, myocardial TG levels in the HFD rat hearts (45.8 ± 9.3 µmol/µg protein) were more than twice those of the control group (21.3 ± 6.9 µmol/µg protein, *P* < 0.05; Figure 2G). A smaller but still significant difference was observed for myocardial free fatty acids, with 0.26 ± 0.07 nmol/µg protein in the HFD group and 0.18 ± 0.04 nmol/µg protein in the control group (*P* < 0.05; Figure 3H). No significant difference was found for TC (Figure 2I).

### 3.3 Mitochondrial morphology changes caused by HFD

The electron microscopic observation of both HFD and control rat cardiac muscle showed marked morphological changes in the HFD samples compared with control samples (Figure 3A,B). The structure of the myocardia from the HFD group showed obscured striation and partial and even complete disappearance of the z line. Ultrastructural analysis of the mitochondria showed that the mitochondrial density was noticeably lower in the HFD‐group myocardia and demonstrated a greater variation in size. Moreover, HFD‐group myocardia also appeared to be bloated and had fewer cristae structures in the inner membrane.

### 3.4 HFD impairs mitochondrial function in regard to energy production

The mitochondrial function in regard to energy production was evaluated by quantification of adenine nucleotide variants in the myocardia. The ATP and ADP concentrations in the myocardia of the HFD group were 1.78 ± 0.45 and 2.03 ± 0.23 µg/mL, respectively, which were significantly lower than those of the control group (2.83 ± 0.40 and 2.50 ± 0.15 µg/mL, respectively, *P* < 0.05; Figure 3C). Conversely, the AMP concentration was significantly higher in the HFD group than in the control group (1.30 ± 0.36 vs 0.78 ± 0.14 µg/mL, respectively, *P* < 0.05; Figure 3C). No significant differences existed between the control and HFD groups in regard to total adenine nucleotide contents (Figure 3C). Consequently, the HFD group had a significantly higher AMP/ATP ratio (0.75 ± 0.04 vs 0.26 ± 0.03, respectively, *P* < 0.05; Figure 3D) and a lower energy charge (0.55 ± 0.05 vs 0.67 ± 0.03, respectively, *P* < 0.05; Figure 3E).

### 3.5 Mitochondrial complex activity is decreased in the cardiac mitochondria of the HFD group

To evaluate the oxidative phosphorylation efficiency of the cardiac mitochondria, maximal activities of complexes I‐III and citrate synthase were measured in cardiac mitochondria from both the control and HFD groups. Mitochondrial complex enzyme activities were found to have decreased dramatically (Figure 3) in the HFD group. The activity dropped from 19.5 ± 5.1 to 6.2 ± 1.3 for complex I (*P* < 0.05; Figure 3F), from 21.9 ± 4.1 to 13 ± 1.4 for complex II (*P* < 0.05; Figure 3G), from 125.3 ± 5.7 to 80.0 ± 8.4 for complex III (*P* < 0.05; Figure 3H), and from 371.2 ± 38.5 to 244.0 ± 35.0 for citrate synthase (*P* < 0.05; Figure 3I).

### 3.6 Decreased mtDNA copy number in the HFD group

**Figure 4:**
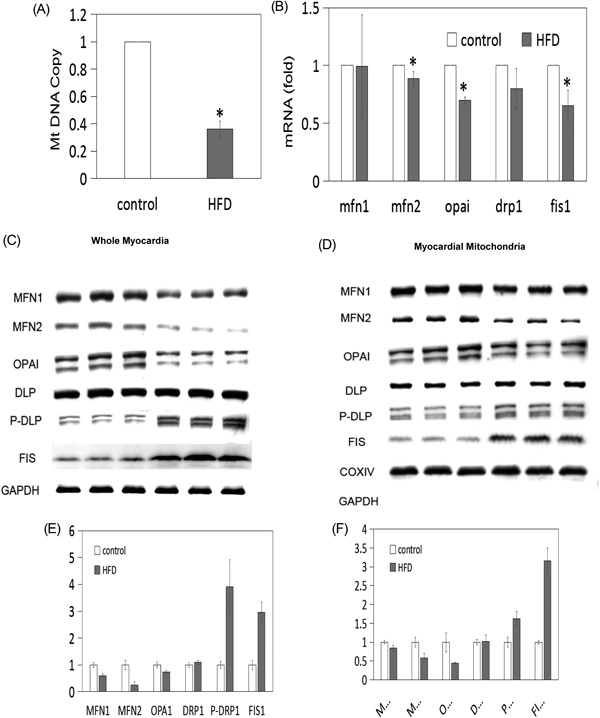
Changes in mitochondrial‐related protein expression in the HFD and control groups. (A) Compared to the control, the mtDNA copy number was lower in the HFD group (*P* < 0.05), suggesting impaired mitochondrial biogenesis. (B) Compared to the control group, the HFD group showed decreased mRNA levels for all of the genes assessed, including the mitochondrial fusion genes mfn1, mfn2, and opa1 and the mitochondrial fission genes drp1 and fis1. (C,D) The protein levels of mitochondrial fusion proteins were markedly decreased in the HFD group, while levels of mitochondrial fission proteins were increased. (E,F) Densitometric quantification of the Western blot results presented in (C) and (D) using GAPDH and COXIV as loading controls. COXIV, cytochrome *c* oxidase subunit 1; GAPDH, glyceraldehyde 3‐phosphate dehydrogenase; HFD, high‐fat diet; mtDNA, mitochondrial DNA

The mtDNA copy number was determined by quantitative PCR. The copy number was lower in the HFD group (39.1 ± 6.1%) than in the control group (*P* < 0.05; Figure 4A), which suggested impaired mitochondrial biogenesis.

### 3.7 Altered expression of genes involved in mitochondrial dynamics

The expressions of genes controlling mitochondrial fusion and fission were analyzed at both the mRNA and protein levels. Compared with the control group, the HFD group showed decreased mRNA levels for all of the assessed genes, including the mitochondrial fusion genes mfn1, mfn2, and opa1 and the mitochondrial fission genes drp1 and fis1 (Figure 4B). Among these genes, mfn2, opa1, and fis1 demonstrated significant differences between the two groups (*P* < 0.05).

The protein levels of these genes in both the whole myocardia and isolated myocardial mitochondria were analyzed by Western blot. In both the whole myocardia and myocardial mitochondrial protein extracts, the mitochondrial fusion proteins MFN1, MFN2, and OPA1 were markedly decreased in the HFD group, while the mitochondrial fission proteins, such as phosphorylated DRP1 and FIS1, were dramatically higher in the HFD group (Figure 4C,D). Densitometric quantification of the Western blot results confirmed that the differences were statistically significant using GAPDH and COXIV as loading controls for the whole myocardia and myocardial mitochondrial samples, respectively (Figure 4E,F).

### 3.8 Analysis of the mitochondrial proteome in cardiac mitochondria

Mass spectrometry quantification of the mitochondrial proteome using iTRAQ labeling showed a widespread decrease in mitochondrial proteins in the HFD group in comparison with the control group. Proteins that were involved in fatty acid oxidation, complex I and V, and mitochondrial fusion, such as acetyl‐coenzyme A dehydrogenase short chain, 3‐ketoacyl‐CoA thiolase, NADH dehydrogenase (ubiquinone) 1α subcomplex subunit 10, NADH‐ubiquinone oxidoreductase chain 4, ATP synthase subunit α, ATP synthase subunit ɣ, mitofusin‐2 protein (MFN2), and others, were all significantly downregulated in the HFD group (Table 3).

**Table 3:** Proteins identified by proteomic analysis

Energy metabolism	Fold change compared with the control
*Fatty acid oxidation*	
Acetyl‐coenzyme A dehydrogenase, short chain	0.55^*^
3‐Ketoacyl‐CoA thiolase	0.31^*^
Hydroxyacyl‐coenzyme A dehydrogenase	0.28^*^
Enoyl‐CoA delta isomerase 1	0.64^*^
Delta(3,5)‐delta (2,4)‐dienoyl‐CoA isomerase	0.57^*^
Long‐chain specific acyl‐CoA dehydrogenase	0.46^*^
*Glucose oxidation*	
Pyruvate kinase	1.67
Dihydrolipoyllysine‐residue acetyltransferase component of pyruvate dehydrogenase complex	0.75
Pyruvate dehydrogenase kinase	1.35
Pyruvate dehydrogenase E1 component subunit β	1.77
*Citric acid cycle*	
Mitochondrial pyruvate carrier 2	0.18^*^
Malate dehydrogenase	0.19^*^
*OXPHOS proteins*	
Complex I	
NADH dehydrogenase (ubiquinone) 1α subcomplex subunit 10	0.58^*^
NADH dehydrogenase (ubiquinone) 1β subcomplex 6 (predicted)	2.25^*^
NADH‐ubiquinone oxidoreductase chain 4	0.36^*^
NADH dehydrogenase (ubiquinone) flavoprotein 1	0.59^*^
Complex II	
Succinate dehydrogenase (ubiquinone) cytochrome *b* small subunit	0.16
Succinate dehydrogenase (ubiquinone) flavoprotein subunit	1.04
Complex III	
Cytochrome *b*‐*c*1 complex subunit 2	0.63
Cytochrome *b*‐*c*1 complex subunit 7	0.37
Cytochrome *b*‐*c*1 complex subunit 8	0.18
Cytochrome *b*‐*c*1 complex subunit 1	0.51
Cytochrome *b*‐*c*1 complex subunit 6	1.42
Cytochrome *b*‐*c*1 complex subunit Rieske	0.63
Complex IV	
Cytochromeacaoxidaseasubunita1	0.2
Cytochrome *c* oxidase subunit 2	0.54
Cytochrome *c* oxidase subunit 3	0.59
Cytochrome *c* oxidase subunit 4 isoform 1	0.45
Cytochrome *c* oxidase subunit 5B	0.49
Cytochrome *c* oxidase subunit 6B1	0.41
Complex V	
ATP synthase subunit α	0.57^*^
ATP synthase subunit ɣ	0.25^*^
ADP/ATP translocase 1	0.27^*^
Electron transfer flavoprotein‐ubiquinone oxidoreductase	0.36^*^
Electron transfer flavoprotein subunit α	0.30^*^
2‐Oxoisovalerate dehydrogenase subunit α	2.39^*^
*Mitochondrial dynamics proteins*	
Mitofusion	
Mitofusin‐1 protein (MFN1)	0.52
Mitofusin‐2 protein (MFN2)	0.48^*^
Dynamin‐like 120 kDa protein (OPA1)	0.64
Mitofission	
Regulator of microtubule dynamics protein 1	1.08
Mitochondrial fission 1 protein (FIS1)	2.2^*^

^*^ Significant changes compared with the control, *P*  < 0.05.

In addition, a small number of proteins were significantly upregulated after HFD treatment, including NADH dehydrogenase (ubiquinone) 1β subcomplex 6, 2‐oxoisovalerate dehydrogenase subunit α, and mitochondrial fission protein FIS1 (Table 3).

## 4 DISCUSSION

Our data suggest that feeding rats a HFD for 28 weeks caused cardiac dysfunction, myocardia hypertrophy, and the accumulation of lipids in the myocardia. A further examination of the myocardial mitochondria revealed widespread abnormalities, including morphological changes, such as smaller size, decreased density, disruption of inner membrane cristae, and functional damage in the form of lower efficiency of energy production and decreased enzyme complex activities. A gene expression analysis suggested that the observed mitochondrial dysfunction might be attributable to the downregulation of genes related to energy generation and mitochondrial dynamics.

Our findings generally agree with previous reports on human subjects that have found a positive correlation between body weight and LV mass, between various body weights and LV wall thickness, and between body weight and LV diastolic chamber size.^15^, ^16^ Moreover, the cardiac function of HFD rats was negatively affected, and parameters of LV function, such as EF and FS, were significantly decreased.^17^

The mechanisms underlying HFD‐induced heart dysfunction are not fully understood, even after decades of extensive research.^7^, ^9^, ^18^, ^19^ It is becoming clear, however, that lipotoxicity plays an important role in this process. The deleterious effects of the accumulation of fatty acids in cardiac and skeletal muscle cells cause mitochondrial dysfunction. In addition to producing energy, mitochondria are also the major sources of ROS. Fatty acids accumulating in the vicinity of mitochondria are vulnerable to ROS‐induced lipid peroxidation, which has subsequent lipotoxic effects on mtDNA, RNA, and proteins of the mitochondrial machinery, leading to mitochondrial dysfunction.^20^ Indeed, we identified a broad detrimental impact of HFD on the myocardia mitochondrial functions in the rats tested. The ATP production and energy charge of the HFD group were significantly lower than those of the control group, which may be a direct consequence of impaired mitochondrial dynamics.

Our results demonstrated that impaired mitochondrial dynamics were clearly involved, as multiple genes related to mitochondrial dynamics were significantly altered by HFD treatment. In particular, mitochondrial fusion genes were downregulated and mitochondrial fission genes were upregulated at both the mRNA and protein levels. Recently, mitochondrial dynamics were evaluated to assess mitochondrial morphology, quality, and abundance in cardiovascular disease.^21^ Fusion connects neighboring depolarized mitochondria and mixes their contents to maintain membrane potential, while fission segregates damaged mitochondria from healthy ones and subjects them to mitophagy or fusion, respectively. Mitochondrial fusion is generally believed to be beneficial for the heart because it consolidates the mitochondria's ability to supply energy.^22^ We speculated that the lipotoxicity leading to myocardia mitochondrial dysfunction and impaired mitochondrial dynamics is involved in HFD‐associated cardiac dysfunction. This lipotoxic damage is not only detrimental to mitochondrial functions via impairment of respiratory enzyme complexes, but is also changes the expression profile of genes involved in mitochondrial dynamics. The observed upregulation of the mitochondrial fission genes P‐DRP1 and FIS1 and the downregulation of the mitochondrial fusion genes MFN1/2 and OPA1 would result in less efficient mitochondria in the tissues of the HFD rat heart and could worsen the energy deficiency present in the myocardium.

Our proteomic data also confirmed the observed alteration in mitochondrial‐dynamics–related gene expression and showed a general decrease in mitochondrial enzymes involved in fatty acid and carbohydrate oxidation as well as respiratory complex proteins, which was consistent with our quantitative PCR and Western blot analyses. In conclusion, a HFD induces myocardia mitochondrial dysfunction and impairs mitochondrial dynamics, which highlights the mitochondria as a prospective therapeutic target for the treatment of HFD‐associated heart disease.

One limitation of this study is that human and rodents have different patterns of fat distribution in the heart. Although many researchers support the notion that rodents and humans are comparable, notable differences have emerged related to fat distribution and function of white adipose tissue. Currently, much of what has been tested in translational research has relied heavily on rodents. Since rodents are the most commonly used model for human obesity, further validation of important differences between rodents and humans are still necessary. Thus, further research is warranted to more carefully define the strengths and limitations of rodents as a model organism in regard to such investigations.

One potential future development of this current research is to further decipher the phenomenon of the so‐called obesity paradox. A mounting body of evidence from epidemiological, clinical, and preclinical studies has shown that fatty acids may be beneficial in certain cardiac disease conditions.^7^ Dhahri et al,^23^ reported that a higher polyunsaturated fatty acid content may lead to different results and a diet rich in omega‐3 polyunsaturated fatty acids and complex carbohydrates could confer lipo‐protection and decrease LV hypertrophy. Moreover, advanced analytical techniques based on mass spectrometry have led to the identification of novel biologically active FA‐derivatives and are currently being used to dissect the entire lipidome.^24^ Our future research will involve feeding animals diets of varying FA components and analyzing the subsequent lipidome and proteome using state‐of‐the‐art technologies to obtain a deeper understanding of fat metabolism in the myocardium and to help discover novel therapeutic targets and biomarkers of HFD‐induced heart disease.

## CONFLICTS OF INTEREST

The authors declare that there are no conflicts of interest.
